# Continuous Renal Replacement Therapy: A Review of Use and Application in Pediatric Hematopoietic Stem Cell Transplant Recipients

**DOI:** 10.3389/fonc.2021.632263

**Published:** 2021-02-26

**Authors:** Lama Elbahlawan, John Bissler, R. Ray Morrison

**Affiliations:** ^1^ Division of Critical Care Medicine, St. Jude Children’s Research Hospital, Memphis, TN, United States; ^2^ Department of Pediatrics, University of Tennessee Health Science Center and Le Bonheur Children’s Hospital, Memphis, TN, United States; ^3^ Department of Pediatrics, St. Jude Children’s Research Hospital, Memphis, TN, United States

**Keywords:** continuous renal replacement therapy (CRRT), renal replacement therapy, hematopoietic (stem) cell transplantation (HCST), fluid overload, pediatrics, outcome

## Abstract

Hematopoietic stem cell transplant (HSCT) is a curative therapy for malignant and non-malignant conditions. However, complications post-HSCT contribute to significant morbidity and mortality in this population. Acute kidney injury (AKI) is common in the post-allogeneic transplant phase and contributes to morbidity in this population. Continuous renal replacement therapy (CRRT) is used often in the setting of AKI or multiorgan dysfunction in critically ill children. In addition, CRRT can be useful in many disease processes related to transplant and can potentially improve outcomes in this population. This review will focus on the use of CRRT in critically ill children in the post-HSCT setting outside the realm of acute renal failure and highlight the benefits and applications of this modality in this high-risk population.

## Introduction

Around 2,000 children received HSCT in 2018, and of these, the majority were allogeneic transplants (1). Children admitted to the intensive care unit (ICU) during their HSCT course tend to have a mortality rate as high as 60% especially if associated with multiorgan dysfunction (2). Acute kidney injury (AKI) commonly occurs in this population during different intervals of the post-transplant course. AKI may progress to acute renal failure requiring renal replacement therapy (RRT) to mitigate the metabolic abnormalities as well as fluid overload. There are various RRT techniques like intermittent hemodialysis, extended hemodialysis, or continuous renal replacement therapy (CRRT). Although there is no evidence that mortality or length of ICU stay differ among the different techniques, CRRT is used often for RRT in the intensive care unit (ICU) as the hemodynamic changes are less pronounced with this modality (3). Continuous modalities are better able to tightly manage fluid balance and offer the ability to reduce the need for significant reductions in the circulating volume during the relative short time of hemodialysis. Furthermore, by prolonging the duration of ultrafiltration, the balance of ultrafiltration and lymphatic return can be better matched to optimize cardiac output. In addition, continuous modalities offer better control of electrolyte fluctuations. For example, swings in potassium can be problematic for patients on medications that exacerbate prolonged Q-T syndrome. Lastly, the continuous removal of metabolic waste products reduces the cellular stress and is thought to improve healing. This review will focus on use of CRRT in critically ill children in the post-HSCT setting outside the realm of acute renal failure and highlight the benefits and applications of this modality in this high-risk population.

### Acute Kidney Injury Post-Hematopoietic Stem Cell Transplant

AKI is relatively common post-HSCT and warrants early intervention to halt or slow down further progression. The reported incidence of AKI varies widely from 20 to 84% secondary to heterogeneity of criteria used to define AKI (**Table 1**) as well as heterogeneity of HSCT regimens and subsequent complications (4–6).

**Table 1:** AKIN, KDIGO, pRIFLE staging criteria for AKI.

AKIN Staging	KDIGO staging	pRIFLE Staging
Stage 1	Stage 1	
Scr:1.5–2.0× Bl OR ≥0.3 mg/dl	Scr:1.5–1.9× Bl OR ≥0.3 mg/dl	R = Risk for renal dysfunction/eGFR by 25%
**UO**: <0.5 ml/kg/h for >6 h	**UO**: <0.5 ml/kg/h for 6–12 h	**UO**: <0.5 ml/kg/h for 8 hr
Stage 2	Stage 2	
Scr: >2–3× from Bl	Scr:2.0–2.9× Bl	I = Injury to the kidney/eGFR by 50%
**UO**: <0.5 ml/kg/h for >12 h	**UO**: <0.5 ml/kg/h for 12–24 h	**UO**: <0.5 ml/kg/h for 16 h
Stage 3	Stage 3	
Scr: >3× from Bl	Scr: 3.0× Bl OR ≥4.0 mg/dl	F = Failure of kidney function/eGFR by 75%
OR RRT	OR RRT	OR eGFR < 35 ml/min/1.73 m 2
	OR eGFR <35 ml/min/1.73 m2	
**UO**: <0.3 ml/kg/h for 24 h	**UO**: <0.3 ml/kg/h for ≥24 h	**UO**: <0.3 ml/kg/h for 24 h
OR Anuria for 12 h	OR Anuria for ≥12 h	OR Anuria for 12 h
		L = Loss of kidney function/Persistent failure >4 weeks
		E = End-stage renal disease/Persistent failure >3 months

AKIN, Acute Kidney Injury Network; KDIGO, Kidney Disease Improving Global outcomes; pRIFLE, pediatric Risk, Injury, Failure, Loss, End-Stage Renal Disease; AKI, acute kidney injury; Scr, serum creatinine; Bl, baseline; UO, urine output; eGFR, estimated glomerular filtration rate.

**Figure 1:**
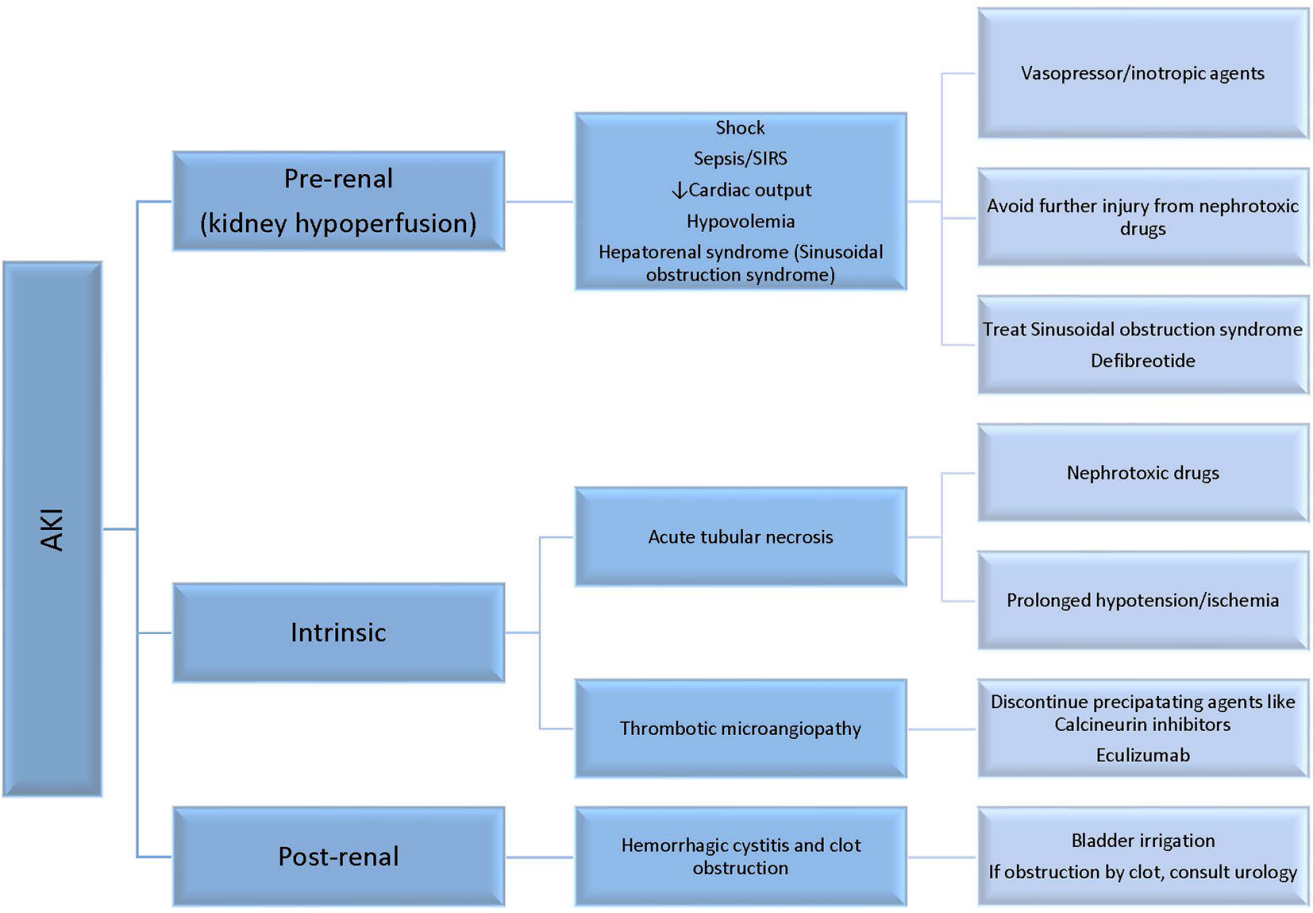
Common etiologies of acute kidney injury (AKI) in children following HSCT.

AKI can occur for several reasons after HSCT (**Figure 1**) such as toxicity of specific conditioning regimens or nephrotoxicity from immunosuppressive and anti-infectious agents, or the setting of pretransplant toxicity especially in children heavily treated with nephrotoxic chemotherapeutic agents. Myeloablative allogeneic HSCT has the highest incidence of AKI compared to nonmyeloablative HSCT or auto-SCT. At 1 month following allogeneic HSCT, the incidence of AKI is 45.7 *versus* 17.1% comparing myeloablative conditioning regimen to reduced toxicity myeloablative conditioning regimen (7). In a large pediatric HSCT cohort of 1,057 patients, 68.2% developed AKI in the first 100 days post-HSCT (6). Stage 3 AKI occurred in 36.6% of the cohort, of which 1/3 required RRT. Risk factors for AKI included older age, transplant from unrelated donors, and sinusoidal obstruction syndrome (SOS). Comparing data from successive decades of 1990–1999 and 2000–2009, the requirement for RRT was similar in the 100 days post-HSCT (8.2 *versus* 8.9%), and children with older age (11–21 years at time of HSCT) or cyclosporin use were more likely to require dialysis (8).

In general, causes of AKI can be secondary to pre-renal factors, intrinsic renal injury at the tubular or endothelial level, and less commonly post-obstructive nephropathy (**Figure 1**). Pre-renal AKI accounts for more than 50% of the cases seen in this population. Pre-renal factors such as hypovolemia can lead to kidney hypoperfusion and AKI if not treated. Kidney hypoperfusion is also encountered in capillary leak syndrome that is relatively common in the post-HSCT course due to endothelial injury and cytokine release, like in engraftment syndrome. In addition, kidney hypoperfusion occurs in the context of multiorgan failure, sepsis, and sinusoidal obstruction syndrome (SOS) (6, 8).

Intrinsic renal disease is encountered as a result of tubular injury induced by nephrotoxic medications or ischemia, or endothelial injury like in thrombotic microangiopathy (TMA). TMA occurs in 10–25% of transplant recipients. Clinical findings of TMA include AKI, hypertension, thrombocytopenia, and microangiopathic hemolytic anemia. In transplant associated-TMA, endothelial injury leads to platelet aggregation and fragmentation of erythrocytes that enhances fibrin deposition and thrombosis in the microcirculation. Many factors can induce endothelial injury in the transplant setting including radiation or chemotherapy during conditioning, calcineurin and mTOR inhibitors, infection, and/or complement dysregulation. Treatment includes elimination of causative agents like sirolimus and calcineurin inhibitors, or appropriate antimicrobial coverage if induced by infection. Recently, eculizumab has shown some efficacy in treatment of transplant associated-TMA (9). Less commonly, AKI can be induced by obstruction such as seen in hemorrhagic cystitis. In these instances, if the clot obstruction is not relieved, AKI may ensue.

In some instances, patients may require CRRT due to progression of AKI. Early management of AKI includes cessation of nephrotoxic medication or dosage adjustment, prevention of fluid overload through judicious use of diuretics, and promotion of renal perfusion by increasing hydrostatic pressure.

CRRT general indications in critically ill children following HSCT are similar to other critically ill children including uremia, hyperkalemia, acidosis, or fluid overload encountered in the context of acute renal failure. In addition, CRRT is used in other disease processes encountered in the post-HSCT phase.

### Use of Continuous Renal Replacement Therapy Post-Hematopoietic Stem Cell Transplant Children for Purposes Beyond Renal Failure

#### Fluid Overload

Fluid overload (FO) is a relatively common indication for CRRT initiation in children post-HSCT. In addition, FO is an independent risk factor for mortality in critically ill children. FO is very common in the post-transplant stage considering the high fluid intake due to the need of multiple medications as well as TPN. Conservative fluid strategy and diuretics are used as the initial approach to prevent FO. However, some patients will fail this approach especially in the presence of capillary leak or low oncotic pressure or high-volume intake. In these situations, CRRT has been utilized in critically ill children with FO with or without the presence of AKI. In a cohort of 51 pediatric stem cell patients, the most common indications for CRRT were FO with electrolyte imbalance (49%), FO alone (39%), and electrolyte imbalance (8%) (10).

Adverse impact of FO in patients post-HSCT occurs even outside the critical care setting. Recently, a grading system for the adverse event of fluid toxicity was described (11). Patients with weight gain ≥10% (grade 2) early during hospitalization had a higher non-relapse mortality. Multiple studies have documented that FO is an independent risk of mortality in critically ill children especially when >15% of baseline weight. A metanalysis that included 44 studies (7,505 children) found a 6% increase in mortality for every 1% increase in FO %. In addition, FO was associated with increased risk of prolonged mechanical ventilation (>48 h) and AKI (12). In a recent multicenter retrospective study that included 68 critically ill children with cancer and post-HSCT (23 patients) on CRRT, patients with FO >10% at CRRT initiation were 6.16 times more likely to die than those with FO ≤10% (13). In another cohort of 36 children post-HSCT with acute respiratory failure due to engraftment, higher FO on day 4 and day 5 of invasive mechanical ventilation course was associated with worse survival (14). Use of CRRT to mitigate the extent of FO has the potential to overcome the adverse effects of FO on mortality and prolonged ICU stay and should be considered early in the ICU course of post-HSCT patients.

#### Septic Shock

The role of CRRT in pediatric post-HSCT patients has received important consideration for some time, and its potential for clinical benefit in shock sepsis is a topic of vigorous discussion (15–20). Rationale for use of CRRT in sepsis is mostly centered around its potential for mitigating volume overload that often accompanies requisite fluid administration for hemodynamic support during sepsis and its putative role for clearing sepsis-associated inflammatory cytokines (19). In this regard, there is developing interest in the use of membranes designed for hydrophobic adsorption of endotoxin and inflammatory cytokines. While several retrospective studies demonstrate safety of various membranes and their efficacy for reducing circulating cytokines, randomized trials have failed to demonstrate reduced mortality (21). Likewise, while early (*versus* late) use of CRRT in sepsis-associated AKI receives considerable debate (19, 20), the recently completed STARRT-AKI trial assessing standard *versus* accelerated initiation of RRT in AKI failed to demonstrate reduction in 90-day mortality with early onset of CRRT (22). In a randomized study of a cohort of 212 patients with septic shock, although high dose hemofiltration (80 cc/kg) reduced cytokine levels significantly compared to the low dose hemofiltration (40 cc/kg), mortality rate was similar in both groups (23). To conclude, current evidence supports the use of CRRT in the setting of sepsis-associated AKI, to manage volume overload and/or metabolic derangement.

#### Sinusoidal Obstruction Syndrome/Veno-Occlusive Disease

Sinusoidal obstruction syndrome (SOS), previously known as hepatic veno-occlusive disease (VOD), can be a life-threatening complication in post-HSCT patients. Sinusoidal endothelial injury plays a central role in the pathogenesis of SOS which is usually induced by cytotoxic therapy or radiotherapy (24). The incidence of SOS in children post-HSCT ranges from 20 to 60% (25, 26). Several diagnostic criteria have been used (modified Seattle and Baltimore criteria), with recent adoption of the European Society for Blood and Marrow Transplantation (EBMT) criteria (27). The EBMT Pediatric Criteria have no limitation for time of onset of SOS. Two or more criteria should be fulfilled for the diagnosis of SOS:

- Unexplained consumptive and transfusion-refractory thrombocytopenia
- Unexplained weight gain on 3 consecutive days despite the use of diuretics, or a weight gain of >5% above baseline value within 72 h
- Increase in bilirubin from baseline on 3 consecutive days, or bilirubin ≥2 mg/dl within 72 h (32% of pediatric patients develop anicteric SOS)
- Hepatomegaly and ascites above baseline.

High mortality rate is reported in SOS when associated with multi-organ dysfunction (MOD) (>80%) (28). Defibrotide is the drug of choice for treatment of SOS in children who have pulmonary or renal dysfunction.

SOS management poses many challenges including a degree of kidney injury secondary to hypoperfusion and vasoconstriction as well as fluid overload. Diuretics are administered either intermittently or continuously to achieve euvolemia. Recently, the consensus report by the Pediatric Acute Lung Injury and Sepsis Investigators (PALISI) and the Supportive Care Committee of Pediatric Blood and Marrow Transplant Consortium (PBMTC) recommended initiation of CRRT when FO progresses despite fluid restriction and diuresis, especially when FO approaches >15% (29). In addition, ascites usually develops secondary to portal hypertension and capillary leak syndrome and may increase intrabdominal pressure. Intrabdominal pressure (IAP), which is usually measured indirectly by bladder pressure, should be monitored closely. Intrabdominal hypertension (IAP >12 mmHg) can lead to abdominal compartment syndrome (IAP >20) which can exacerbate the pre-existing kidney injury (30). In these situations, ascites should be drained promptly.

In a pooled analysis of three studies that included patients with SOS and MOD after HSCT (total of 651 adult and pediatric patients), 22% had dialysis dependence and the estimated survival rate at day 100 was 33% (31). When CRRT was used in six children who developed SOS after HSCT, four survived with mortality rate of 34%; one progressed to ESRD and underwent kidney transplantation. Citrate anticoagulation use was safe with no subsequent complications of bleeding or citrate accumulation (32).

#### Chimeric Antigen Receptor-T Cell Therapy

Chimeric antigen receptor (CAR) T cell therapy has emerged as a novel cellular immunotherapy for the management of hematologic malignancy. However, cytokine release syndrome (CRS) is a potential complication of this therapy and can be fatal in severe cases. The massive cytokine release induces a systemic inflammatory response that can progress to multiorgan failure including AKI if untreated. In a cohort of children with acute lymphoblastic leukemia treated with an anti-CD19 CAR T cell therapy, 46% developed AKI with grade 3–4 CRS (33). Similarly, the cumulative incidence of any grade AKI by day 100 was 30% in a cohort of 46 adults with non-Hodgkin lymphoma treated with CAR-T cell therapy (34). Current management strategies for serious CRS toxicity include anti-cytokine therapy, most commonly tocilizumab, and corticosteroids. Theoretical benefits of CRRT in this setting include cytokine removal, fluid overload management, as well as AKI support. The benefit of cytokine removal by CRRT is controversial, thus, there is not enough evidence to support the use of CRRT solely for that purpose. However, if multiorgan failure ensues despite current standard management, CRRT can be utilized as a supportive modality for severe AKI. In addition, capillary leak associated with CRS will result in fluid overload, thus, increase risk of mortality. CRRT can be initiated to overcome fluid overload if other management approaches fail.

#### Acute Liver Failure/Hyperammonemia

Hepatic dysfunction is relatively common post-HSCT and can be secondary to conditioning regimens, total parental nutrition (TPN), iron overload, infection/sepsis, SOS, or GVHD of the liver. Furthermore, hyperammonemia can be life threatening and is associated with hepatic encephalopathy, cerebral edema, intracranial hypertension that can result in herniation and death. Renal replacement therapy reduces ammonia levels and has been used in infants with urea cycle defects or patients with liver failure. In a large cohort of 1,186 adults with acute liver failure (ALF), use of CRRT resulted in a 37.9% reduction in serum ammonia level (comparing day 3 to day 1) compared to 18.6% in patients not receiving RRT (35). In addition, 21‐day transplant free survival improved. Severe hyperammonemia (>140 μmol/L) was prevented in 84% of a cohort of 62 patients with ALF after CRRT commencement (36). CRRT should be considered when serum ammonia level exceeds 150–200 μmol/L despite medical management, especially in the presence of other risk factors like hepatic encephalopathy, cerebral edema, and intracranial hypertension (35, 37).

One of the concerns with CRRT use in patients with ALF is anticoagulation with citrate. The risk of citrate accumulation is potentially higher due to the impaired citrate metabolism by the liver. A metanalysis that included 10 studies that examined the use of citrate in adults with ALF, the risk of citrate accumulation in patients with liver dysfunction wasn’t significantly higher compared to those without liver dysfunction (38). In addition, pH, serum lactate, and total Ca/ionized Ca ratio was comparable in both groups. Furthermore, the filter lifespan increased significantly with citrate use. Therefore, the use of citrate is safe in these patients and prolongs the life span of the filter. Heparin use in these patients is compromised by reduced hepatic antithrombin III production with SOS.

Another important consideration in children with cerebral edema is the potential drop in serum sodium that may be triggered by starting CRRT. Maintaining relative hypernatremia (keeping Na in 150 range) is a common approach in children with cerebral swelling. In such cases, serum sodium levels should be monitored closely, and ideally a standard protocol implemented for adjusting the replacement/dialysate fluid sodium concentration appropriately to avoid fluctuations in serum osmolarity.

#### Nutrition

Children post-HSCT are at risk of malnutrition due to the decline of their nutritional status. This can occur secondary to the side effects of chemotherapy agents during conditioning like vomiting, diarrhea, anorexia, and mucositis, or/and secondary to complications like infections and acute graft-*versus*-host-disease (aGVHD) (39). Malnutrition has negative impact on risk of infection in pediatric cancer and on overall survival (OS), and relapse risk in adult HSCT (39). Malnutrition status is further exacerbated by critical illness. Critically ill children experience malnutrition and protein-energy wasting due to catabolism during the stress-response state.

CRRT can facilitate achievement of daily caloric intake goals by increasing fluid intake without the concern of fluid overload. In addition, initiation of CRRT can significantly reduce intestinal wall edema, which enhances feeding tolerance and the ability to absorb nutrients.

Nonetheless, significant amounts of amino acids, vitamins, micro- and macro-elements are cleared due to their small molecular weights and have to be supplemented. Protein intake should be increased by 0.2 g/kg ideal body weight or by 25% (sometimes more if the clearance is high) when patients are on CRRT, and total protein intake should be in the range of 1.7–2.5 g/kg IBW. Fats are not removed by CRRT; thus, no adjustments for fat intake are needed while patients are on CRRT. Vitamins B1, B6, and C and folate are lost in the effluent and should be supplemented. Vitamin B12 and fat-soluble vitamins are not removed in effluent, thus, supplementation is not required (40).

### Continuous Renal Replacement Therapy Technique and Practical Considerations

#### Continuous Renal Replacement Therapy

**Figure 2:**
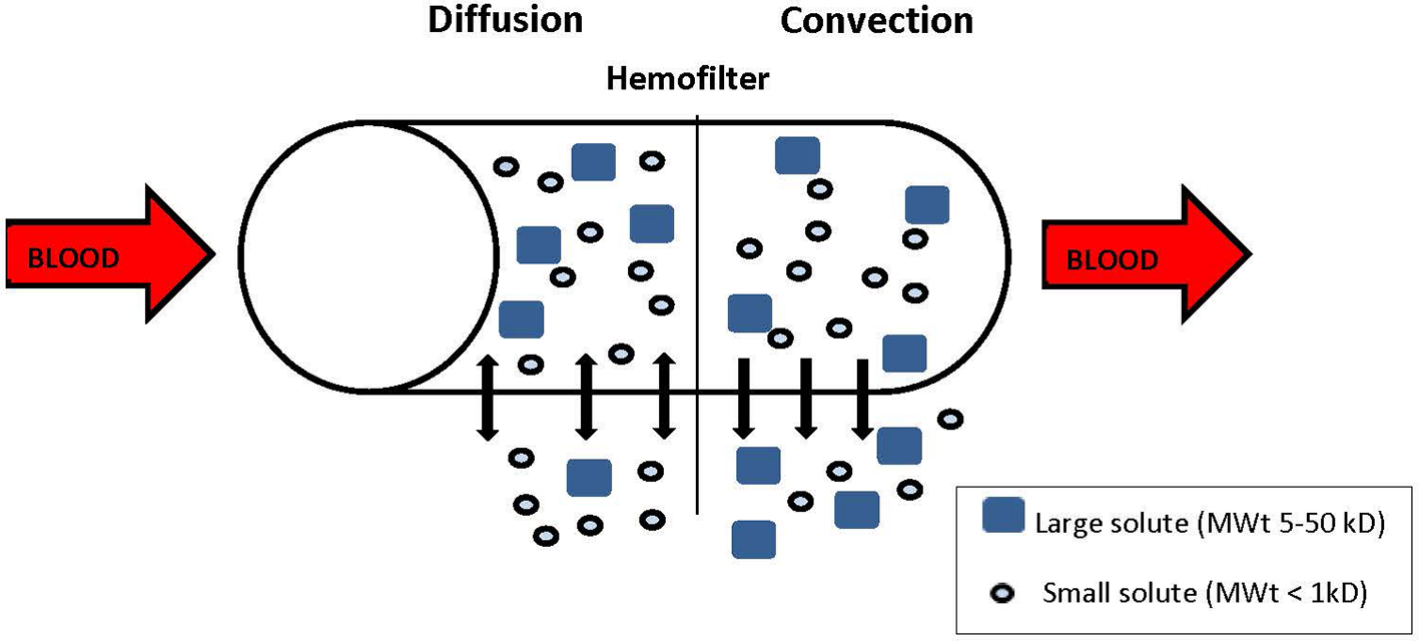
Solute removal. Diffusion: solutes diffuse down their concentration gradient, Convection: solutes are dragged by the flow of plasma water (solvent drag). Small solutes are removed by diffusion and convection, larger solutes are removed by convection.

During CRRT, blood is withdrawn from the venous circulation, pumped through a circuit with a semipermeable membrane (or filter) back to the venous circulation, *via* a large caliber double lumen central catheter. The success of CRRT is dependent on the vascular access, and upper central access is greatly preferred to reduce issues of recirculation. There are different modalities of CRRT, depending on the mode of solute clearance. The two main solute clearance modalities are diffusion and convection (**Figure 2**). Diffusion is the movement of solute across a semipermeable membrane down a concentration gradient. Dialytic modes like continuous veno-venous hemodialysis (CVVHD) or continuous veno-venous hemodiafiltration (CVVHDF) use diffusion for solute clearance and the dialysate solution runs countercurrent to the blood flow. Clearance depends on the concentration gradient, the size and the protein binding of the solute, and the surface area of the filter. Diffusion favors clearance of low molecular weight solutes like urea (60 Da) and creatinine (113.12 Da), compared to those of higher molecular weight like cystatin C (13 kDa) and cytokines (6–70 kDa). Convection involves the movement of water and dissolved solutes (solvent drag) across a semipermeable membrane due to a pressure gradient. Middle and large molecules can be cleared through convection depending on the membrane porosity. Hemofiltration modalities like continuous veno-venous hemofiltration (CVVH) and CVVHDF use convection for solute clearance, and replacement solutions are infused before (pre-) or after (post-) the filter. CVVHDF also includes a diffusive component to further facilitate clearance of smaller molecules more than larger ones. For hemofiltration, solute clearance is determined by the fluid replacement/ultrafiltrate rate across the semipermeable membrane, and the location of the replacement fluid infusion (pre- or post- filter).

#### When?

Timing of initiation of CRRT may optimize outcome, but the impact on mortality is still controversial. In the recent STARRT-AKI trial that included 3,019 critically ill adults with AKI, patients were randomized to either an accelerated RRT strategy (initiated within 12 h) or a standard strategy. Risk of death at 90 days was similar in both groups and survivors of the accelerated RRT strategy had higher risk of adverse events as well as dependence on RRT (22). However a prior randomized trial (231 critically ill patients with AKI), mortality was lower in the early RRT group compared to the delayed initiation group (39 *versus* 54% respectively) (41). Although early *versus* delayed initiation of RRT is controversial, there is ample of evidence in the literature to support use of CRRT when FO >10% and even more so when FO >15%. Therefore, FO of >10–15% is a reasonable timing for initiation of CRRT in this population.

#### How?

Hemofiltration removes larger molecules more effectively, but the clearance is different between pre- and post-filter replacement. The clearance (K) in post-dilution CVVH is calculated as the product of the sieving coefficient (S) and the ultrafiltration rate (Quf). The sieving coefficient is best thought of as the concentration of the marker in the filtrate divided by the concentration in the blood.

K=S×Quf

For small solutes such as urea and creatinine, the sieving coefficient is effectively a value of one, making small solute clearance in post-dilution CVVH equal to the ultrafiltration rate. Pre-filter replacement is not the same because the clearance in pre-dilution CVVH must account for the fact the blood concentrations are reduced by the pre-filter dilution of the blood. Understanding the clearance in this case can be managed by multiplying the clearance K by a “dilution factor” consisting of the ratio of the blood water flow rate (Qbw) to the sum of Qbw and the replacement fluid rate (Qrf).

$$K=S\times Quf\times \frac{Qbw}{\left(Qbw+Qrf\right)}$$

The addition of dialysate (CVVHDF) can further facilitate the clearance of small molecules.

Effective CRRT hinges on vascular access. In children, upper access is by far preferable because it reduces the issues of recirculation.

With respect to the choice of CRRT modality, the practice is highly variable and there is not enough evidence to favor one modality over the other. In a cohort of pediatric stem cell patients on CRRT, CVVHD was used in 43%, followed by CVVH in 37%, and then CVVHDF in 20% (10). In a metanalysis that included 19 randomized controlled trial, mortality was similar in the hemofiltration *versus* the hemodialysis modality in patients with acute kidney injury (42).

Drug clearance is altered by CRRT and depends on the drug’s molecular weight, degree of protein binding, volume of distribution, and its sieving coefficient. Drugs that are primarily protein bound are cleared minimally during CRRT. It is important to check blood levels for drugs if available (like vancomycin) and adjust dosage accordingly.

#### Dose

CRRT dose is the volume of blood purified per unit of time. It is usually estimated by the effluent rate (expressed in cc/kg/hr) (21). In a metanalysis that included patients in six randomized studies, intensive CRRT (>35 cc/kg/hr) did not reduce the risk of mortality compared to the less intensive CRRT (<35 cc/kg/hr) (43). Another metanalysis with 2,970 patients found 90 day mortality to be similar between the high-dose or low-dose hemofiltration (44). However, many studies fail to consider where the replacement fluid is running and the effective dose, complicating interpretation if the data presented.

Furthermore, the prescribed dose is often not the delivered dose due to interruptions that occur during CRRT (like circuit clotting, scheduled filter change). To compensate for the difference between the prescribed and delivered dose, KDIGO recommends 25–30 ml/kg/h prescription dose in order to achieve a delivered dose of 20–25 ml/kg/h, and minimal therapy interruptions (45).

#### Anticoagulation

Anticoagulation is used during CRRT to prevent filter clotting and is achieved often with either heparin anticoagulation or RCA-CRRT. During RCA-CRRT, about 30–70% of the administrated citrate (192 Da) is removed by circuit depending on the blood and fluid flow rates. The remaining citrate is metabolized by the liver. Calcium is chelated during RCA, therefore, continuous calcium drip is infused and titrated according to patient ionized calcium (iCa) levels. Citrate toxicity can occur if citrate accumulates and should be suspected in the context of worsening metabolic acidosis, a decrease in systemic iCa, or a total calcium/iCa ratio >2.5:1.

In a recent randomized trial in 638 critically ill adults on CRRT that compared RCA *versus* systemic heparin anticoagulation, median filter life span was significantly higher with RCA *versus* heparin group (47 *vs* 26 h). In addition, bleeding complication was significantly less in citrate *versus* heparin group (5.1 *versus* 16.9%) (46). A recent review that included seven studies with 535 participants, major bleeding was observed in 22/1,000 with RCA *versus* 102/1,000 when unfractionated heparin used (47). Therefore, citrate is a better option for anticoagulation in children with HSCT who are at higher risk of bleeding secondary to thrombocytopenia or coagulopathy. Clinicians may choose not to use pharmacologic anticoagulation in patients at high risk of bleeding, but the risk of circuit clotting and interruption of treatment is higher. In a cohort of 103 patients with liver failure on CRRT, filter life was significantly longer in the RCA group *versus* the no-anticoagulation group (>72 *versus* 39.5 h) (48). However, bleeding incidence was similar in both groups (5.1% in the RCA group *versus* 4.3% in the no-anticoagulation group), suggesting that RCA use is a safe option in patients with a higher risk of bleeding. Non-pharmacologic measures that affect lifespan of the filter include modality; CVVHDF modality has 44% less risk of filter clotting compared to the CVVHD modality (49). Saline flushing at the prefilter site didn’t increase lifespan of filter in patients who didn’t receive anticoagulation during CRRT (50).

#### Infants <10 kg

CRRT in infants <10 kg is challenging for multiple reasons. To run CRRT efficiently, large size vascular catheter is usually required, at least 7 French, which may be difficult to insert in a small infant. Catheter location is also critical, and upper lines are far preferable to femoral lines to limit recirculation. In addition, the volume needed to prime the CRRT circuit is relatively large (92 and 165 ml). The smaller CRRT circuit (Prismaflex™ M60) has a higher risk of bradykinin release syndrome. The Prismaflex™ HF20 [extracorporeal volume (ECV) = 60 ml] is available outside USA (51). In addition, these larger machines require higher blood rates that can potentially lead to massive clearance rates.

CRRT circuit is usually primed with packed red blood cells (PRBC) in these infants to reduce morbidity. However, PRBC are very concentrated, acidic, hyperkalemic, and hypocalcemic. The hematocrit of 60 of the PRBC can be reduced by a mix of 1:1 PRBC: 5% albumin which will achieve an estimated hematocrit of 35%. To normalize PH and electrolytes in PRBC, CRRT circuit that was primed with blood can be dialyzed prior to the initiation of CRRT using a physiological dialysate solution (52). Another helpful approach in infants at the time of change of the CRRT circuit is the rapid exchange technique. Rapid exchange of CRRT is a sequential technique that transfers citrated blood from one CRRT machine to another machine connected in series, thus, eliminating the need for blood priming of the new CRRT circuit (53). Few machines with smaller CRRT circuits (like CARPEDIEM) under development and investigation will allow the use of a smaller vascular catheter and lower blood flow rates.

Survival rate has been historically lower in children <10 kg (43 *vs.* 64% in children >10 kg; p < 0.01) (54). In a recent cohort of 71 infants with weights <10 kg on CRRT, survival rate was 57.7%. After CRRT initiation, survivors had less need for vasopressor support, lower oxygen requirement, and lower FO% (55).

### Complications

CRRT is an invasive therapy with potential complications and increased resources utilization. Insertion of a dedicated vascular central line catheter is essential, therefore, there is introduced risk of infection and thrombosis. In addition, electrolyte disturbances are common (like hypophosphatemia, hypomagnesemia, hypocalcemia, alkalosis), thus, serum electrolyte levels need to be checked and followed closely. Hypothermia is a potential complication and external warming may be required. Thermal loss during CRRT may mask fever and delay recognition of infection, so other parameters of infection should be monitored closely. Hypotension may occur at any time during CRRT and may necessitate the reduction of fluid removal rate or initiation/escalation of vasopressor support.

### Outcome

Mortality in children post-HSCT requiring RRT is reported to be as high as 52–65% (**Table 2**) (17). Recently, ICU survival has improved but long-term survival is still poor (16, 59). In a cohort of 122 children requiring RRT post-HSCT, 1-year overall survival between 1990 and 1999 was 11% with improved survival of 23% during the more recent period between 2000 and 2009 (8). In a recent HSCT cohort of 1,102 children, RRT was used in 11.9% of admissions with 51.9% mortality at ICU discharge (60).

**Table 2:** Outcome of CRRT summary in pediatric stem cell patients.

Study	# of patients	Comorbidities	Modality of CRRT	Factors associated with mortality	PICU survival (%)
Michael et al. (56)	26 11 on CRRT	MV: 77% MV: 100% on CRRT	CVVHD (2 patients) 9 not specified	MV Prism 2 score ≥10 <10% FO: better survival	18
Flores et al. (10)	51	MV: 73%	CVVH (37%) CVVHD (43%) CVVHDF (20%)	MV Higher Paw at end of CRRT	45 35 in MV cohort
Rajasekaran et al. (16)	29	MV: 82% Vasopressors: 76%	CVVHD	Lower PaO2/FiO2 at 48 h Higher CRP at end of CRRT Neutropenia at end of CRRT	33 >6m: 6%
Elbahlawan et al. (57)	30	MV: 100% Vasopressors: 43%	CVVH (13%) CVVHD (87%)	Higher Paw at 48 h post CRRT (p = 0.099)	17
Lombel et al. (58)	21	MV: 86% Vasopressors: 57	CVVHD CVVHDF	%FO: Not associated with mortality	61 6 m: 29%
Gilbert et al. (59)	68 (HSCT on MV) 20 (on CRRT)	MV: 100%	CVVHD	Respiratory dysfunction Liver dysfunction	>35d: 10%
Raymakers-Janssen et al. (13)	68 mixed (cancer and HSCT) 23 (HSCT)		CVVHD CVVHDF CVVHF	FO Vasoactive support	54% in all cohort 39% in HSCT cohort

CRRT, continuous renal replacement therapy; CVVH, continuous veno-venous hemofiltration; CVVHD, continuous veno-venous hemodialysis; CVVHDF, continuous veno-venous hemodiafiltration; MV, mechanical ventilation; FO, fluid overload; Paw, mean airway pressure; CRP, C-reactive protein; HSCT, hematopoietic stem cell transplant.

It is unknown whether one modality of CRRT is superior to another or whether timing of initiation of CRRT changes outcome. Randomized trials are needed to outline the best practice to improve survival.

## Conclusion

CRRT has many useful applications in critically ill children post-HSCT. Fluid overload >15% is associated with higher risk of mortality, therefore, CRRT should be considered early if other measures to control FO fail. Beyond registry data of outcomes of different approaches, a detailed, well-designed trial with precise calculated and measured clearances of small, moderate, and large molecular markers is desperately needed to improve the care of the children post-HSCT requiring CRRT. To facilitate this, a change in the way of thinking so that the dose of ultrafiltration and clearance are separately defined and measured delivery would significantly improve the understanding of how to improve patient outcome.

## Author Contributions

LE contributed to planning, writing, and editing the manuscript. JB contributed to writing and editing the manuscript. RM contributed to writing and editing the manuscript. All authors contributed to the article and approved the submitted version.

## Funding

This was funded by ALSAC.

## Conflict of Interest

The authors declare that the research was conducted in the absence of any commercial or financial relationships that could be construed as a potential conflict of interest.
